# Configurable topological photonic polycrystal based on a synthetic hybrid dimension

**DOI:** 10.1093/nsr/nwaf107

**Published:** 2025-03-24

**Authors:** Tianyue Li, Mengjiao Liu, Jin Qin, Jianzheng Ren, Jiahao Hou, Yang Liu, Xing Yang, Hongchen Chu, Yun Lai, Shuming Wang, Jian-Hua Jiang, Che Ting Chan, Shining Zhu

**Affiliations:** National Laboratory of Solid State Microstructures, School of Physics, Nanjing University, Nanjing 210093, China; Collaborative Innovation Center of Advanced Microstructures, Nanjing University, Nanjing 210093, China; Department of Physics, The Hong Kong University of Science and Technology, Hong Kong 999077, China; National Laboratory of Solid State Microstructures, School of Physics, Nanjing University, Nanjing 210093, China; Collaborative Innovation Center of Advanced Microstructures, Nanjing University, Nanjing 210093, China; National Laboratory of Solid State Microstructures, School of Physics, Nanjing University, Nanjing 210093, China; Collaborative Innovation Center of Advanced Microstructures, Nanjing University, Nanjing 210093, China; State Key Laboratory for Artificial Microstructure and Mesoscopic Physics, School of Physics, Peking University, Beijing 100871, China; National Laboratory of Solid State Microstructures, School of Physics, Nanjing University, Nanjing 210093, China; Collaborative Innovation Center of Advanced Microstructures, Nanjing University, Nanjing 210093, China; School of Physical Science and Technology, Collaborative Innovation Center of Suzhou Nano Science and Technology, Soochow University, Suzhou 215006, China; National Laboratory of Solid State Microstructures, School of Physics, Nanjing University, Nanjing 210093, China; Collaborative Innovation Center of Advanced Microstructures, Nanjing University, Nanjing 210093, China; School of Physics and Technology, Nanjing Normal University, Nanjing 210023, China; National Laboratory of Solid State Microstructures, School of Physics, Nanjing University, Nanjing 210093, China; Collaborative Innovation Center of Advanced Microstructures, Nanjing University, Nanjing 210093, China; National Laboratory of Solid State Microstructures, School of Physics, Nanjing University, Nanjing 210093, China; Collaborative Innovation Center of Advanced Microstructures, Nanjing University, Nanjing 210093, China; Key Laboratory of Intelligent Optical Sensing and Manipulation, Ministry of Education, Nanjing 210093, China; School of Physical Science and Technology, Collaborative Innovation Center of Suzhou Nano Science and Technology, Soochow University, Suzhou 215006, China; Suzhou Institute for Advanced Research, University of Science and Technology of China, Suzhou 215123, China; Department of Physics, The Hong Kong University of Science and Technology, Hong Kong 999077, China; National Laboratory of Solid State Microstructures, School of Physics, Nanjing University, Nanjing 210093, China; Collaborative Innovation Center of Advanced Microstructures, Nanjing University, Nanjing 210093, China; Key Laboratory of Intelligent Optical Sensing and Manipulation, Ministry of Education, Nanjing 210093, China

**Keywords:** topological photonics, synthetic dimensions, pseudospin-valley coupling, domain walls, photonic integrated circuits

## Abstract

Topological photonic structures exhibit resilience to defects, allowing unidirectional light flow and promoting the development of robust devices with large information processing capacities. However, the diversity of topological boundary modes is typically governed by bulk-edge correspondence, which limits multidimensional multiplexing and the integration density of next-generation photonic systems. Here, we present a polycrystal approach based on domain wall engineering to configure multi-band dispersion in a synthetic hybrid dimension by utilizing orientation freedom. As a prototype, we demonstrate that an all-dielectric platform for hybrid topological polycrystalline photonic integrated circuits can support up to eight edge channels and four corner modes via pseudospin-valley Hall effect, empowering controllable directionality of multi-frequency and spinful channels with highly localized performance. Our findings reveal a photonic architecture that significantly advances the on-chip integration of topological photonics, offering valuable potential for future information processing technologies across optical and microwave frequencies.

## Teaser

Hybrid Photonic polycrystals with a synthetic hybrid dimension enable versatile control of edge channels and corner modes in multiple frequency bands.

## INTRODUCTION

Configurable artificial photonic materials offer new opportunities to study and control topological phases [1,2]. The photonic analogue of the quantum Hall effect (QHE) exhibits topological resilience, with edge states that remain immune to defects and disorder through suppressed backscattering [3,4]. Later, time-reversal-symmetric topological photonic insulators with hexagonal lattice symmetry enabled diabolic Dirac-like dispersions [5], giving rise to the quantum spin Hall effect (QSHE) [6,7] and quantum valley Hall effect (QVHE) [8–10], which provide more freedom in light manipulation and unlock higher-order topological states [11,12]. Additionally, synthetic dimensions are introduced to allow the exploration of topology beyond observable space [13], transcending the traditional constraint where physical dimensions are limited to three-dimensional geometric spaces. This concept includes coupling artificial lattice parameters, such as frequency [14,15], time [16] and spatial modes [17], or simply changing the structural parameters [18–20], which is particularly effective for reducing high-dimensional information into 2D photonic platforms, empowering versatile phenomena, such as rainbow trapping [21,22] and dislocation states [23], and broadening the scope of topological physics.

Despite various theoretical and experimental successes in topological photonics, practical applications, such as realizing one-way waveguides in photonic integrated circuitry (PIC) [24,25], are essential to meet the growing demand for large-scale information processing and to avoid fabrication-induced disorder and defects [26]. However, the number of available channels in topological waveguides (TWGs) is typically constrained by bulk-edge correspondence and often requires multiple lattice periods, which limits the diversity of edge states and results in a relatively large device footprint. Although TWGs with large Chern numbers can support more interface modes [27], they are heavily dependent on specific wavevectors and lack flexibility in tuning dispersion.

An alternative approach lies in domain-wall (DW) engineering by applying hybrid topological photonic crystals (HTPCs), which facilitate interactions between distinct topological phases at their interfaces. For instance, HTPCs based on the quantum anomalous Hall effect (QAHE) and QVHE can produce multi-frequency boundary channels by breaking time-reversal ($\mathcal{T}$) and parity ($\mathcal{P}$) symmetries [28]. Nevertheless, the weak magneto-optical effects required to break $\mathcal{T}$-symmetry pose challenges for scalability, particularly in extending TWGs from lightwave to microwave regimes. Moreover, the precise etching of dielectric structures into metallic boundaries remains a significant obstacle to large-scale fabrication. Therefore, straightforward, multidimensional and dispersion-configurable topological photonic structures based on an all-dielectric platform are essential, aiming to simultaneously increase the number of channels and enhance their multiplexing capabilities, empowering high-quality localized optical routing and developing high-order topological modes.

Here, we report a type of configurable topological photonic polycrystal (TPPC) based on a synthetic hybrid dimension, spanning a 1D momentum space and a 2D synthetic orientation space. Distinct from QAHE-QVHE-based HTPCs, these microstructures consist of all-dielectric orientated elliptical elements relying on the coupling of pseudospin-valley Hall effect (PVHE). This framework provides a universal platform to simultaneously manipulate multiple frequencies, spins and group velocities of dispersion by effectively downgrading the 3D band hypersurfaces into 2D topologically protected modes. We propose a scalable perturbation theory for rapidly calculating and tuning the photonic bandgap and introduce a localized quality metric based on the local density of states (LDOS) to evaluate the performance of such hybrid topological devices. As a proof of concept, we design hybrid topological polycrystalline photonic integrated circuits (HTP-PICs) and experimentally demonstrate both multi-band edge and corner modes with a high-contrast localized representation along the interface. Our work significantly advances on-chip control over topological phases across a wide range of frequencies and spins, thereby creating new opportunities for dense photonic integration from classical to quantum regimes.

## RESULTS

**Figure 1.**
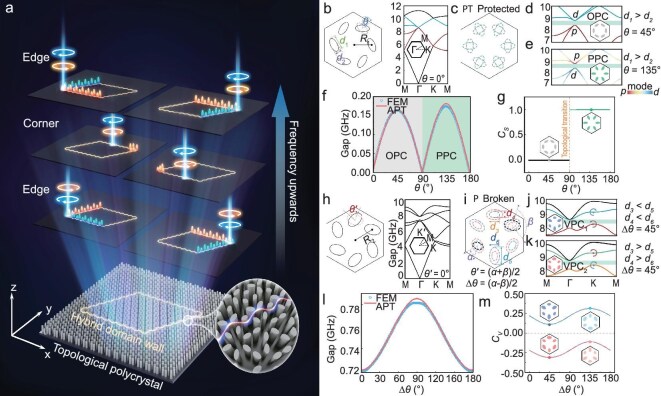
The topological photonic polycrystal (TPPC) applied to photonic integrated circuitry (PIC) and its detailed composition. (a) Schematic illustration of HTP-PIC, which exhibits edge and corner states. The right and upper edges support TESs with the same spin-momentum locking at different frequencies, while the left and lower edges support TESs with opposite spin-momentum locking at different frequencies. (b–e) The *C*_6_-symmetric hexagonal lattice exhibits band degeneracies at the Γ point near 8 GHz (b) and the lattice varies as the orientation angle *θ* changes, where the angle increases in the clockwise direction (c), and the band structures of two variant cells with *θ* = 45° (OPC) and *θ* = 135° (PPC) are shown in (d) and (e), where *d* and *p* modes are labeled. (f) The relevance between gapped frequency and rotation angles *θ* through FEM simulation and APT calculation. (g) The numerically calculated *C_S_* as a function of *θ*. (h) The *C*_6_-symmetric hexagonal lattice (left) and its band structure showing degeneracies at K points around 8 GHz (right). (i) *C*_3_-symmetric cell with broken parity symmetry $\mathcal{P}$ with changing orientation angle evolved from the lattice in (h). (j, k) The band structures of two variant cells with different changes of geometric parameters. VPC_1_ has bigger ellipse at the top and VPC_2_ has smaller ellipse at the top, and circular arrows denote the valley-polarized at the K point of the corresponding bands. (l) The relevance between gapped frequency and rotation angles Δ*θ* through FEM simulation and APT calculation. (m) The numerically calculated *C_V_* of the two VPCs as a function of Δ*θ*.

The all-dielectric TPPC designed for PIC applications can be exemplified by a 2D photonic crystal (PhC) consisting of periodic hexagonal lattices of dielectric ellipses with a relative dielectric constant of *ε_r_* = 11.8 embedded in air, as shown in Fig. 1a, giving rise to multi-band and spin-dependent edge and corner modes. The magnified inset clearly shows the PVHE-coupled DWs, with periodic lattices on either side exhibiting distinct sizes and orientations, which are categorized as 6-fold (C_6_) and 3-fold (C_3_) rotational symmetries, corresponding to the QSHE and QVHE, respectively. The left panel of Fig. 1b details the C_6_-symmetric lattice, where the dielectric ellipse is defined by its orientation angle *θ* (0 ≤ θ ≤ *π*), major axis *d*_1_ and minor axis *d*_2_. The eigenstates are labeled by the Bloch wave vector (*k_x_, k_y_*), forming a 2D parameter space, with *θ* treated as a synthetic dimension. Since all three parameters are periodic and mutually independent, they collectively define a 3D parameter space (*k_x_, k_y_, θ*), within which one can study the eigenfrequency *ω_n_* (*k_x_, k_y_, θ*) for a given band number *n*. Based on the Wu-Hu model [29], these parity-time $\mathcal{P}\mathcal{T}$-protected lattices exhibit a 4-fold degenerate Dirac cone dispersion at the Γ point in the transverse magnetic (TM) mode, as illustrated in the right inset of Fig. 1b. At the moment of bandgap closure, *θ* is defined as the initial angle 0°, with the clockwise direction designated as positive. The coupling strengths within the lattice are governed by the interplay between intra-cellular and inter-cellular coupling. In our model, coupling is modulated by rotating all six elliptical pillars in the left diagram of Fig. 1b from the solid-line configuration to the dashed-line layout by the angle *θ*, and thereby opening the degeneracy, as illustrated in the two cases shown in Fig. 1c. Examples of their band structures are presented in Fig. 1d and e, corresponding to *θ* = 45° and *θ* = 135°, respectively. For convenience, we label these configurations ordinary PhC (OPC) and pseudospin PhC (PPC), reflecting the occurrence of band inversion. Detailed structural parameters and their corresponding eigenmodes are provided in Supplementary Data A.

Focusing on the Γ point, we fix *k_y_* and investigate how the orientation angle influences the gap, effectively reducing the subspace (*k_x_, θ*) in the 2D PhC to a 1D system. The bandgap as a function of *θ* is plotted in Fig. 1f, calculated using a scalable method known as angle-perturbation theory (APT) (details in Supplementary Data B). Specifically, the rotation of the dielectric ellipse is equivalent to a perturbation in the material's dielectric constant, affecting spatial modes and their frequencies [30]. This is analogous to tuning the mass term of the lattice during adiabatic evolution to manipulate the dispersion [31,32]. The method is versatile, applicable to various models, and enables rapid quantification of dispersion. As the angle *θ* increases, the bandgap gradually widens, reaching a first peak at *θ* = 45°. Beyond this, the gap begins to close, only to reopen and reach a second peak at *θ* = 135°, before finally closing again. The results from finite element method (FEM) simulations align closely with those derived from APT calculations.

The topology can be further characterized by spin Chern number *C_S_*, which can be obtained by integrating the Berry curvature ${\mathrm{\Omega }}( {\vec{k}} ) = \nabla \times B( {\vec{k}} )$ over the first Brillouin zone (FBZ) as:


(1) \begin{eqnarray*}
{C}_s = \frac{1}{{2{\mathrm{\pi }}}}\mathop \int \nolimits_{FBZ} {\mathrm{\Omega }} ( {\vec{k}} ){d}^2\vec{k}.
\end{eqnarray*}

The zero and non-zero spin Chern numbers indicate topological trivial and non-trivial phases, respectively [33]. The numerically calculated *C_S_* as a function of *θ* is displayed in Fig. 1g, and finds *C_S_* = ±1 for pseudospin up and down states when *θ* is between 90° and 180°, guaranteeing the existence of pseudospin edge states; while *C_S_* = 0 for those states when *θ* is between 0° and 90°, indicating topological trivial phases. Topological transitions are observed at *θ* = 0 (or 180°) and *θ* = 90°, as indicated by the dashed lines in Fig. 1g, which stand for the closure of the band gap at the Γ point. In another way, through a ***k***·***p*** theory, the spin Chern numbers of pseudospin-up (*C*_+_) and pseudospin-down states (*C*_-_) can be evaluated as:


(2) \begin{eqnarray*}
{C}_ \pm = \pm \frac{1}{2}\left[ {{\mathrm{sgn}}\left( M \right) + {\mathrm{sgn}}\left( B \right)} \right],
\end{eqnarray*}

where $M = \frac{{\varepsilon _d^0 - \varepsilon _p^0}}{2}$, with $\varepsilon _d^0$ and $\varepsilon _p^0$ being the eigenfrequencies of quadrupole and dipole eigenmodes at the ${\mathrm{\Gamma }}$ point, and *B* is determined by the diagonal elements of the second-order perturbation term of effective Hamiltonian which is typically negative [34]. Consequently, the spin Chern numbers will be *C_S_* = ±1 when $\varepsilon _d^0 < \varepsilon _p^0$ as for PPC and ${C}_ \pm = 0$ when $\varepsilon _d^0 > \varepsilon _p^0$ as for OPC.

We now analyze the lattice configuration shown on the left side of Fig. 1h, with its band structure depicted on the right. This structure features a Dirac cone at the K (K’) point due to double degeneracy, which is independent of the lattice orientation *θ*′. To lift this degeneracy, representing a standard configuration for the QVHE, the parity symmetry $\mathcal{P}$ of the lattice must be broken. This leads to the lattice in Fig. 1i, composed of two sets of dashed dielectric ellipses of different sizes, exhibiting C_3_ symmetry. The major and minor axes of the ellipses are marked as *d*_3,5_ and *d*_4,6_ respectively, with their orientation angles defined as *α* and *β* which increase clockwise. We define *θ*′ = (*α* + *β*)/2 (0 ≤ θ ≤ π) and Δ*θ* = (*α* − *β*)/2 (0 ≤ Δ*θ* ≤ *π*). Taking *θ*′ under the configuration in Fig. 1b as the initial position (*θ*′ = 0°), different configurations can theoretically be achieved by varying Δ*θ*. Thus, the parameter space for valley photonic crystals (VPCs) can be defined as (*k_x_, k_y_*, Δ*θ*). Figure 1j and k show two types of VPCs with fixed Δ*θ* = 45°, namely VPC_1_ and VPC_2_, which feature opposite layouts, corresponding to distinct valley degrees of freedom at the K (K') point in the fourth and fifth bands of high-frequency modes (see Supplementary Data A for detailed geometric parameters and eigenmodes). Figure 1l illustrates the dispersion in the (*k_x_*, Δ*θ*) subspace at the K point, with results from both the APT and FEM methods. This confirms that the rotation of the ellipse does not close the bandgap but still influences the eigenfrequency, resulting in oscillatory behavior as Δ*θ* changes.

To further characterize the valley-Hall topology in parameter space, the VPCs exhibit non-zero and oppositely distributed Berry curvatures in the K and K′ regions. The Chern numbers of the K and K′ valleys, *C*_K_ and *C*_K′_, can be obtained by restricting the integration region in Equation (1) to the half-Brillouin zone corresponding to the K and K′ regions, respectively. The valley Chern number is then defined as *C_V_* = *C*_K_ − *C*_K′_, which quantifies the topology of VPCs. Notably, the valley Chern number is not always an integer but varies continuously with changes in structural parameters, such that 0 < |*C_V_*|< 1 [35]. We numerically calculated the valley Chern number for two types of C_3_-symmetric lattices, with Δ*θ* varying from 0° to 180°, as shown in Fig. 1m. The results indicate that the VPCs consistently exhibit topologically non-trivial phases (*C_V_* ≠ 0), ensuring the presence of valley-dependent edge states. Furthermore, a pair of mirror-symmetric lattices are found to have valley Chern numbers of equal magnitude but opposite signs, reflecting different valley-polarized properties, which will support edge states at their interface.

**Figure 2.**
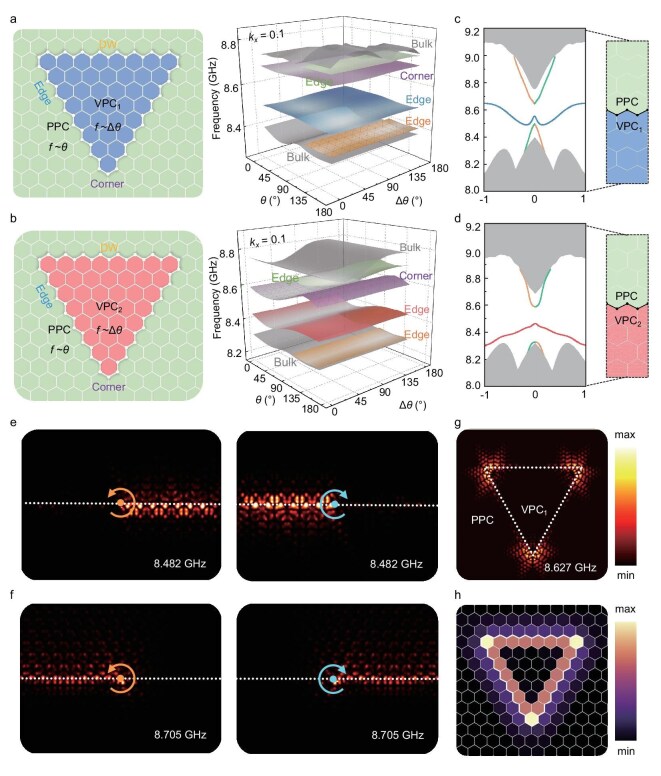
Hybrid topological states in superlattice-based HTP-PIC. (a, b) Schematic of TPPC composed of VPC_1_ (a) and VPC_2_ (b) surrounded by PPC. The right panels show the dispersion of the bands of the corresponding configuration in hybrid dimensions as a function of (*θ*, Δ*θ*) at *k_x_* = 0.1. (c, d) Projected band structures of the supercell combining VPC_1_ (c) or VPC_2_ (d) and PPC at Δ*θ* = 45°. The shaded regions represent the bulk states, whereas the solid lines within the bulk bandgap are the dispersion of the TES. (e, f) Simulated unidirectional propagation of light triggered by LCP and RCP sources at 8.482 GHz (e) and 8.705 GHz (f) for the configuration in (c). (g, h) Field distributions of the corner states (g) and localized quality *Q^L^* distributions (h) in HTP-PIC formed by VPC_2_ nested with PPC.

After discussing two types of topological features within their respective synthetic dimensions, we now demonstrate that topological DW engineering can also be realized in a synthetic hybrid parameter space formed by combining *θ* and ∆*θ*. In the left panels of Fig. 2a and b, we present the TPPC constructed by periodically separately nesting VPC_1_ and VPC_2_ within the PPC structure. Based on the preceding analysis, VPC_1,2_ are associated with ∆*θ*, while the PPC is related to *θ*. When these components interact, they form a hybrid DW with zigzag boundaries at their interfaces due to PVHE coupling. Under these conditions, the system remains periodic only along the *k_x_*, thus defining a 3D hybrid synthetic space (*k_x_, θ*, ∆*θ*). The dispersion of this hybrid dimension as a function of (*θ*, Δ*θ*) at *k_x_* = 0.1 is shown on the right panels of Fig. 2a and b, where six hypersurfaces are clearly observed. Among these are two bulk profiles adjacent to the low- and high-frequency edge regions, one edge profile in the middle, and a corner mode embedded between the two edge regions.

We then construct a supercell combining VPC_1,2_ at Δ*θ* = 45° and PPC to analyze the hybrid edge modes, as shown in Fig. 2c and d. It is apparent that the upper and lower edge bands exhibit pseudospin characteristics similar to those described in references [36,37]. The valley polarization caused by QVHE is sandwiched between these edge bands. Interestingly, due to the different configurations of VPC_1_ and VPC_2_, the group velocities of the valley-polarized edge bands are reversed. The rest of the band structures with other orientated cases can be found in Supplementary Data C. If it is triggered by left-handed circular polarization (LCP) and right-handed circular polarization (RCP) sources, taking the situation of Fig. 2c as an example, Fig. 2e and f result in the opposite propagating directions of edge states at 8.482 GHz and 8.705 GHz by counterclockwise and clockwise symbolled excitation, respectively. Intriguingly, the topological edge states (TESs) excited by the upper and lower frequencies exhibit opposite chiralities, revealing a chiral anomaly in the original spin-momentum locking. The field distributions for other layouts are detailed in Supplementary Data D. However, this phenomenon is absent in certain cases in Fig. S3, which preserve dual-band spin-momentum locking. Notably, the valley edge dispersion in Fig. 2c presents a W shape, resulting from the geometric influence of the VPCs at the DW, which does not alter the bulk topology or mix different valley features [38].

Figure 2g illustrates the corner modes for VPC_1_ nested with PPC, where the field is localized at three corners of the DWs and exhibits exponential decay from the inner region to the outer edges, reflecting a 0D mode distribution. Figure 2a and b present the corner modes of other TPPCs and their eigenfrequencies under different orientation angles, revealing subtle variations related to the lattice tuning angle. Additionally, we calculated the eigenstates for HTP-PICs with OPC and VPC coupling, as detailed in Supplementary Data E. Since OPC is topologically trivial, only VPC-induced TESs exist. To further describe the localized quality of both TESs and topological corner states (TCSs), we have conducted a special treatment of the hybrid eigenmodes within the bandgap. We define a quantitative metric termed ‘localized quality’ *Q^L^* which integrates the local density of states (LDOS) for the target frequency (see methods). A visual representation of *Q^L^* across the lattice is provided in Fig. 2h. The *Q^L^* of edge and corner cells is in quite evident contrast with that of bulk cells far from the boundaries, which suggests the existence of hybrid TESs and TCSs, respectively. Note that *Q^L^* at the corner of DW further indicates that the hybrid TCSs primarily originate from the C_3_-induced VPC. Meanwhile, those TPPC surrounded by OPC within VPCs do not exhibit hybrid TESs after calculating *Q^L^* (see Supplementary Data E). Through this criterion, we can manipulate the eigenfrequencies within the bandgap by controlling the orientation of dielectric pillars. Consequently, the action of rotating them can be employed to modulate and configure the LDOS within the bandgap, which is a critical assessment for laser sources. Meanwhile, Fig. 2a and b also indicate the robustness of the corner states against variations in the orientation of the dielectric ellipse. The reason for the formation of TCSs for non-hybrid PhC can be determined by analyzing the distribution of Wannier centers [39] (Supplementary Data F). However, in the TPPC, the PPC and VPC have a distinct $\mathcal{P}$ that prevents the clear definition of Wannier centers, resulting in an irregular distribution. For this reason, Supplementary Data F shows a numerical calculation of the spectral charge for non-hybrid PhC with perfect conductor boundary (PEC) conditions and hybrid PhC to demonstrate the existence of corner states. Contrary to systems that exhibit only high-order QSHE or QVHE, relying on nearest-neighbor coupling, all configurations of TPPC exhibit TCSs. This indicates that the unconventional TCSs in VPC-embedded PPC originate from long-range coupling [40], unveiling novel phenomena associated with hybrid TCSs.

**Figure 3.**
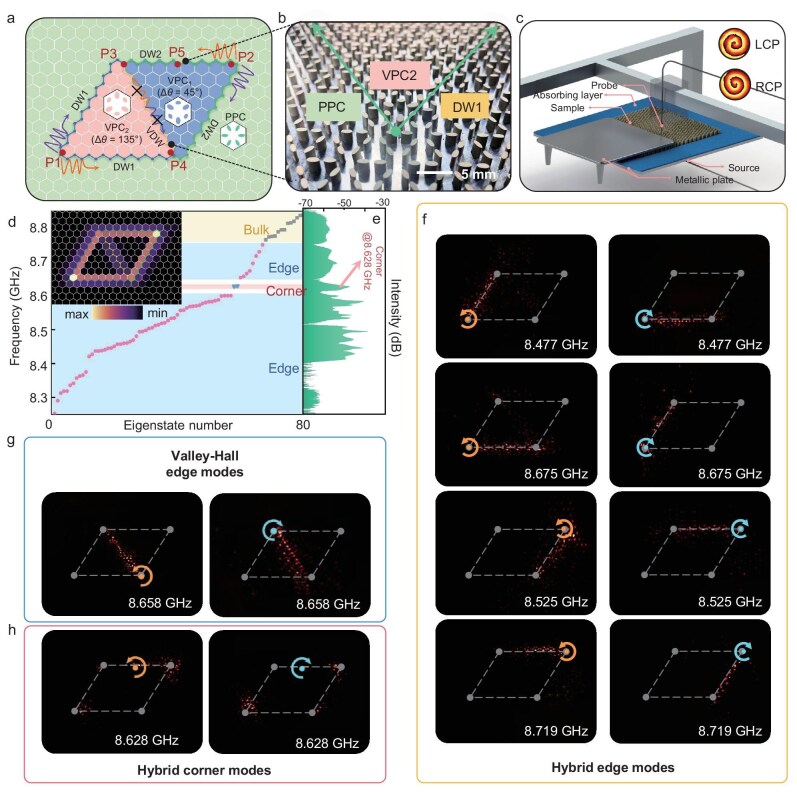
Observation of hybrid TESs and TCSs in symmetry-maintained HTP-PIC. (a) Schematic layout of the HTP-PIC formed by VPC_1_ with Δ*θ* = 45° and VPC_2_ with Δ*θ* = 135° surrounded by PPC. (b) A partial snapshot of DW1 of the HTP-PIC sample. (c) Schematic diagram of the near-field probing set-up used to characterize the sample. (d) Numerically calculated eigenvalues of symmetrical HTP-PIC. The bulk, edge and corner states are represented by squares, dots and inverted triangles, respectively. The inset panel shows the *Q^L^* distribution. (e) The experimentally measured transmission. (f–h) Experimental observation of hybrid TESs at the interfaces of VPC and PPC (f), valley-polarized TESs at the interface of VPC_1_ and VPC_2_ (g), and two hybrid TCSs (h), excited by LCP and RCP sources denoted by counterclockwise and clockwise circular arrows, respectively.

To practically harness TESs and TCSs, we designed the configuration as illustrated in Fig. 3a to form an HTP-PIC and then fabricated finite-sized PhC by carefully depositing alumina ceramic material onto a metal plate. It consists of PPC surrounding VPC_1_ with Δ*θ* = 45° and VPC_2_ with Δ*θ* = 135°, respectively, and a part of the sample is depicted in Fig. 3b. To analyze the sample, we employed pump–probe and near-field detection with the experiment set-up shown in Fig. 3c. The HTP-PIC is precisely aligned using a high-precision stepper motor and carefully covered with a metal plate, which is kept at an exact distance of 0.5 mm above, ensuring the creation of an ideal PEC environment. We initiated a vortex-like chiral source as the input, and used a metal probe to collect the signal (see details in the experiment set-up section). The numerical calculation of the eigenstates of HTP-PIC along with *Q^L^* distribution, as shown in Fig. 3d, reveals the emergence of TESs (indicated in dots) and TCSs (inverted triangle) between the bulk states (square) from 8.25 to 8.85 GHz. The experimentally measured transmission, as depicted in Fig. 3e, indicates that the LDOS between the eigenstates is significantly lower than that of the bulk, edge and corner states, aligning well with the numerical simulations (see Supplementary Data H for the details of the transmission measurement).

The hybrid TESs at 8.477 GHz are obtained through the excitation of LCP and RCP when the source is positioned at the bottom-left corner of the sample (P1). Upon adjusting the frequency to 8.675 GHz, another set of TESs can be acquired. Noticeably, the set of TESs in the higher frequency (the second row in Fig. 3f) propagate counterclockwise (clockwise) when excited by LCP (RCP), showing spin-momentum locking. As for the set of TESs in the lower frequency (the first row in Fig. 3f), the TESs show opposite spin-momentum locking, where TESs propagate clockwise (counterclockwise) when excited by LCP (RCP). Consequently, for a source with a certain direction, the TES in the higher frequency and lower frequency propagate in opposite directions, which can be used for frequency multiplexing. By relocating the source to P2, the chiral TESs with spin-momentum locking and opposite spin-momentum locking are observed at frequencies of 8.719 GHz and 8.525 GHz. Additionally, at the other two ports P3 and P4, the valley-polarized TESs can be excited at 8.658 GHz. This set of dual-band chiral TESs represents a multi-band topological routing. Furthermore, we identified a hybrid TCS within the bandgap-free region, as displayed in Fig. 3e, concentrated near a central frequency of 8.628 GHz with field distributions shown in Fig. 3h (corresponding simulation results are provided in Supplementary Data I). By separately exciting LCP and RCP at the centers of the upper DWs (P5), a pair of TCSs with a 60° feature can be realized, as VPC_1_ is symmetrically mirrored across the Valley DW (VDW) relative to VPC_2_. Consequently, this symmetry-maintained HTP-PIC configuration ensures that they share the same frequencies of TCSs. Besides, due to spin-orbit interactions, chiral-related 120° large angles are excited but do not represent TCSs resulting from topological defects at those locations. The reason for this can be found in Supplementary Data J.

**Figure 4.**
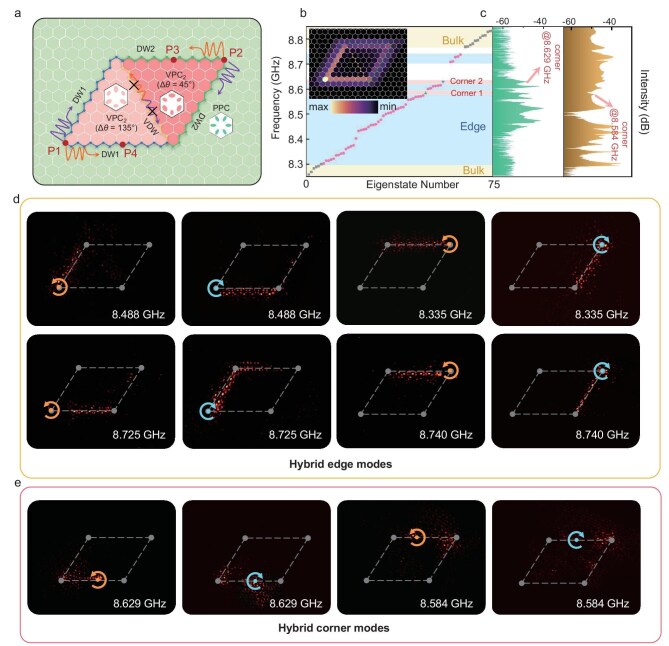
Observation of hybrid TESs and TCSs in symmetric-antisymmetric HTP-PIC. (a) Schematic layout of the HTP-PIC formed by VPC_2_ with different Δ*θ* surrounded by PPC. (b) The numerically calculated eigenvalues of antisymmetric HTP-PIC, where the upward- and downward-pointing triangles indicate corner states with different eigen-frequencies, and the inset panel shows the *Q^L^* distribution. (c) The experimentally measured transmission when exciting the source at P1 and P2, respectively. (d) Experimental observation of eight hybrid TESs at the interfaces of VPC and PPC. (e) Experimental observation of dual-band hybrid TCSs when exciting the HTP-PIC at P3 and P4, respectively.

To realize dual-band TCSs, VPC_2_ with different Δ*θ* are nested HTP-PIC with the same $\mathcal{P}$ but different orientation, to fabricate a symmetric-antisymmetric HTP-PIC, as shown in Fig. 4a, with an antisymmetric configuration of the DWs. In this case, the valley-dependent channel is closed as the two regions have valley Chern numbers of the same sign. Figure 4b depicts the simulated eigenstates and the *Q^L^* distribution in this circumstance, while the corresponding experimental transmission intensities are depicted in Fig. 4c. Similarly, by exciting the sources at P1 and P2, we can totally identify eight hybrid TESs, as shown in Fig. 4d. Notably, at 8.488 GHz and 8.335 GHz, two pairs of TESs are observed, and the field is truncated when propagating from one DW to the other due to frequency shifts caused by different Δ*θ*. Near 8.725 GHz and 8.740 GHz, TESs in the higher frequencies are obtained. As can be seen, the set of TESs in the higher frequency (the second row in Fig. 4d) propagate counterclockwise (clockwise) when excited by LCP (RCP), showing spin-momentum locking. As for the set of TESs in the lower frequency (the first row in Fig. 4d), the TESs show the same spin-momentum locking at the right and upper boundaries (DW2) while showing the opposite spin-momentum locking at the left and lower boundaries (DW1), where TESs propagate clockwise (counter-clockwise) when excited by LCP (RCP). Additionally, the TCSs are found near 8.629 GHz and 8.584 GHz, respectively. We excited the HTP-PIC at P3 and P4 to obtain the field distributions shown in Fig. 4e, with corresponding simulation results provided in Supplementary Data I. Compared with Fig. 3h, dual-band TCSs are realized due to different topological properties of the edges adjacent to the two 60° corners.

## CONCLUSION

We present a strategy for configuring an all-dielectric topological photonic polycrystal (TPPC) framework with pseudospin-valley coupling domain walls based on a synthetic hybrid dimension. This framework exhibits the coexistence of dual-band chiral topological edge and corner states, manifesting in both boundary channels and localized modes.

Furthermore, we design and fabricate two prototype devices for hybrid topological polycrystalline photonic integrated circuits, supporting multi-band, multi-path channels with chirality, where the frequency allocation can be efficiently configured using our proposed APT-based approach. The performance of these devices is evaluated through localized quality metrics *Q^L^*, providing a reliable assessment of their effectiveness. This approach leverages multi-band chiral edge channels to realize on-chip logic gates [41], couplers [42] and dense optical communications [43], while also enabling the development of multi-frequency lasers with ultra-small mode volumes and diverse corner modes [44]. Furthermore, the concept of orientation-dependent photonic lattices facilitates the reconfiguration and reprogramming of active topological devices [45–47]. We envision exploring the non-linear effect of TPPCs to further enhance their multimode and multi-frequency capabilities [48,49], paving the way for significant applications in both classical and quantum phenomena.

## METHODS

### Simulation

The commercial software COMSOL MULTIPHYSICS is employed for the numerical simulations of the samples in this work. As our primary focus is on the TM mode, we employ three pairs of Floquet periodic boundary conditions in a hexagonal lattice to obtain the band structure of the lattice. When simulating the supercell, we designate the in-plane boundaries parallel to the propagation direction as PEC conditions, and the boundaries perpendicular to the propagation direction are set as Floquet periodic boundaries. As for HTP-PIC, we implement scattering boundary conditions, which are applied to the boundaries parallel to the interfaces of the two PhCs. To introduce a source of pseudospin excitations in the boundary and corner states, we first obtain the out-of-plane flow, resulting in the excitation electric field through vortex-like phase profile.

### Localized quality methodology

The concept of localized quality can be derived from the LDOS of PhC. By integrating the LDOS within the bandgap, the mode number for each lattice can be extracted. This count, marked as localized quality (*Q^L^*), is a variant of fractional spectral charge in the target bandgap, which parallels the quantum of charge defined by the fundamental electron charge *e* in electronic systems, where it represents the electron filling from the intrinsic state to the bandgap. We have engineered each C_3_ symmetric lattice composed of all VPC lattices to perform the eigenfrequency *f*_i_ of the model and the normalized wave functions *φ*_i_ for each mode by using FEM simulation. Consequently, the LDOS for the *j*th cell can be represented as [50]:


(3) \begin{eqnarray*}
{\rho }_j\left( {{f}_i} \right) = \mathop \sum \limits_i \int {\left| {{\varphi }_{j,i}\left( {{\bf r}} \right)} \right|}^2d{{\bf r}}.
\end{eqnarray*}

For photonic systems, Equation (3) should be rewritten as:


(4) \begin{eqnarray*}
{\rho }_j\left( {{f}_i} \right) = \mathop \sum \limits_i \int {\varepsilon }_r\left( {{\bf r}} \right){\left| {{E}_{z,i}\left( {{\bf r}} \right)} \right|}^2d{{\bf r}},
\end{eqnarray*}

where ${\varepsilon }_r( {{\bf r}} )$ is the relative dielectric constant and **r** denotes the position vector. The electric field wave function for the *i*th eigenstate is represented by *E_z,i_*. Thus, the localized quality of the *j* th lattice can be deduced by summing the LDOS for the *m*-th to *n*-th eigenstates under the target frequency:


(5) \begin{eqnarray*}
Q_j^L = \mathop \sum \limits_{i = m}^n {\rho }_j\left( {{f}_i} \right).
\end{eqnarray*}

### Microwave experiment

The LDOS and local field distribution of PhC are obtained through a microwave near-field measurement system comprising a vector network analyzer (KEYSIGHT N5224B) and a 2D near-field scanning platform. In the experiment, modulation of the excitation source and collection of field data are achieved by connecting a measurement antenna and an excitation antenna array to the measurement port and excitation port of the vector network analyzers, respectively. During the scanning process, the PhC sample is placed in a parallel-plate waveguide apparatus, with a distance of 6.5 mm between the top and bottom metal plates of the waveguide, much smaller than half the wavelength of the incident wave. The measurement of the dielectric coefficient of PhC composed of elliptic rods can be found in Supplementary Data K. This ensures the uniformity of the electric field along the normal direction of the metal plates inside the waveguide. The measurement antenna is fixed at the bottom of the top plate of the waveguide, measuring the z-component of the electric field at the top of the sample (covering 8.00 to 9.00 GHz). In the experiment, there is an extremely thin air gap (∼0.5 mm) between the cylindrical structures and the top metal plate (measurement antenna). This allows the PhC sample to move smoothly horizontally under the drive of a stepper motor until the entire scanned plane is covered (scan step accuracy of 2 mm). The excitation antenna array consists of three linear antennas connected by a power divider, with phase delays of 0, 2π/3 and 4π/3, respectively, fixed at specific positions on the aluminum plate at the bottom of the PhC. The spacing of 4.5 mm between these three antennas, much smaller than the wavelength of the incident wave, ensures the excitation of pseudospin.

## Supplementary Material

nwaf107_Supplemental_File
