# Supplementary material for: Configurable topological photonic polycrystal based on a synthetic hybrid dimension
Source: Natl Sci Rev. 2025 Mar 24;12(6):nwaf107. doi: 10.1093/nsr/nwaf107 (PMC12153716; doi:10.1093/nsr/nwaf107)
Supplement: nwaf107_Supplemental_File [file nwaf107_supplemental_file.docx]

Supplement for

**Configurable Topological Photonic Polycrystal Based on Synthetic Hybrid Dimension**

Tianyue Li, Mengjiao Liu, Jin Qin *et al.*

*Corresponding author. Email: wangshuming@nju.edu.cn; jhjiang3@ustc.edu.cn; zhusn@nju.edu.cn

**This PDF file includes:**

Sections A to K

Figs. S1 to S12

**Section A. Parameters of lattices and their corresponding eigen-modes**

The corresponding parameter settings related to Figure 1 in the main text are illustrated in Figure. S1. Fig. S1a and Fig. S1b, displaying the structural parameters of the OPC and PPC lattices induced by QSHE, with values *d*_1_ = 2.50 mm, *d*_2_ = 1.20 mm, and *R*_1_ = 0.667 mm. Fig. S1c to Fig. S1f presents four unit cells of VPC with *R*_2_ = 0.715 mm. By transforming the six-fold rotational symmetry C_6_ into a three-fold rotational symmetry C_3_, we obtained two types of ellipses with major and minor axes *d*_3_, *d*_4_ and *d*_5_, *d*_6_, where *d*_3_ = 0.215 mm, *d*_4_ = 0.143 mm, *d*_5_ = 0.285 mm, and *d*_6_ = 0.179 mm for VPC_1_ in Fig. S1c and Fig. S1e, and *d*_3_ = 0.285 mm, *d*_4_ = 0.179 mm, *d*_5_ = 0.215 mm, and *d*_6_ = 0.143 mm for VPC_2_ in Fig. S1d and Fig. S1f. The lower panels show the corresponding eigenmodes at the bands of interest.

**Section B. The establishment of angular perturbation theory (APT)**

Generally, slight adjustments to the shape of resonant materials or the introduction of small dielectric or metallic components are utilized to fine-tune resonance modes. The cavity perturbation method is a practical technique for this purpose, operating under the assumption that the actual fields within a perturbed cavity, featuring minor changes in shape or material, do not significantly deviate from those within an unperturbed cavity. Adapting from this theory, we proposed the angular perturbation theory which explores the relation between the eigenfrequency and the rotation angle 𝜃 of our dielectric scatter. If $\vec{E_{0}}$ and $\vec{H_{0}}$ are the fields of the unperturbed photonic crystal (PhC), and $\vec{E_{0}}$, $\vec{H_{0}}$ are the fields of the perturbed PC, the Maxwell’s equations can be written for the two cases as:

$$\begin{aligned} \nabla\times\vec{E}_{0}=-i\omega_{0}\mu\vec{H}_{0}\#\left( 1 \right) \end{aligned}$$

$$\begin{aligned} \nabla\times\vec{H}_{0}=i\omega_{0}\epsilon\vec{E}_{0}\#\left( 2 \right) \end{aligned}$$

$$\begin{aligned} \nabla\times\vec{E}=-i\omega\left( \mu+\Delta\mu\right)\vec{H}\#\left( 3 \right) \end{aligned}$$

$$\begin{aligned} \nabla\times\vec{H}=i\omega\left( \epsilon+\Delta\epsilon\right)\vec{E}\#\left( 4 \right) \end{aligned}$$

where 𝜔_0_ is the resonant frequency of the original cavity and 𝜔 is the resonant frequency of the perturbed cavity, multiply the conjugate of (3) by $\vec{H}$ and multiply (4) by $\vec{E_{0}^{*}}$, to get:

$$\begin{aligned} \vec{H}\cdot\nabla\times\vec{E}_{0}^{*}=i\omega_{0}\mu\vec{H}\cdot\vec{H}_{0}^{*}\#\left( 5 \right) \end{aligned}$$

$$\begin{aligned} \vec{E}_{0}^{*}\cdot\nabla\times\vec{H}=i\omega\left( \epsilon+\Delta\epsilon\right)\vec{E}_{0}^{*}\cdot\vec{E}\#\left( 6 \right) \end{aligned}$$

Subtracting these two equations and using the vector identity that $\nabla\cdot\left( \vec{A}\times\vec{B} \right)=\vec{B}\cdot\nabla\times\vec{A}-\vec{A}\cdot\nabla\times\vec{B}$ gives:

$$\begin{aligned} \nabla\cdot\left( \vec{E}_{0}^{*}\times\vec{H} \right)=i\omega_{0}\mu\vec{H}\cdot\vec{H}_{0}^{*}-i\omega\left( \epsilon+\Delta\epsilon\right)\vec{E}_{0}^{*}\cdot\vec{E}\#\left( 7 \right) \end{aligned}$$

Similarly, multiply the conjugate of (2) by $\vec{E}$, and multiply (1) by $\vec{H}$, we can get:

$$\begin{aligned} \vec{E}\cdot\nabla\times\vec{H}_{0}^{*}=-i\omega_{0}\epsilon\vec{E}_{0}^{*}\cdot\vec{E}\#\left( 8 \right) \end{aligned}$$

$$\begin{aligned} \vec{H}_{0}^{*}\cdot\nabla\times\vec{E}=-i\omega\left( \mu+\Delta\mu\right)\vec{H}_{0}^{*}\cdot\vec{H}\#\left( 9 \right) \end{aligned}$$

Subtracting these two equations and using vector identity gives:

$$\begin{aligned} \nabla\cdot\left( \vec{E}\times\vec{H}_{0}^{*} \right)=-\text{i}\omega\left( \mu+\text{∆}\text{μ} \right)\vec{H}_{0}^{*}\cdot\vec{H}+i\omega_{0}\epsilon\vec{E}_{0}^{*}\cdot\vec{E}\#\left( 10 \right) \end{aligned}$$

Now, by adding (10) and (7), integrate over the volume 𝑉_0_, and use the divergence theorem to obtain

$\begin{matrix} \int_{V_{0}} \nabla\cdot\left( \vec{E}_{0}^{*}\times\vec{H}+\vec{E}\times\vec{H}_{0}^{*} \right)dv=\oint_{S_{0}} \left( \vec{E}_{0}^{*}\times\vec{H}+\vec{E}\times\vec{H}_{0}^{*} \right)\cdot d\vec{s}=0 \\ =i\int_{V_{0}} \left( \left[ \omega_{0}\epsilon-\omega\left( \epsilon+\Delta\epsilon\right) \right]\vec{E}_{0}^{*}\cdot\vec{E}+\left[ \omega_{0}\mu-\omega\left( \mu+\Delta\mu\right) \right]\vec{H}_{0}^{*}\cdot\vec{H} \right)dv \end{matrix}$ (11)

where the surface integral is zero because $\hat{n}\times\hat{E}=0$ on 𝑆_0_. Rewriting above equation as follows:

$$\begin{aligned} \frac{\omega-\omega_{0}}{\omega}=\frac{-\int_{V_{0}} \left( \Delta\epsilon\vec{E}\cdot\vec{E}_{0}^{*}+\Delta\mu\vec{H}\cdot\vec{H}_{0}^{*} \right)dv}{\int_{V_{0}} \left( \epsilon\vec{E}\cdot\vec{E}_{0}^{*}+\mu\vec{H}\cdot\vec{H}_{0}^{*} \right)dv} \#\left( 12 \right) \end{aligned}$$

This result shows that any increase in 𝜖 or 𝜇 at any point in the cavity will change the resonant frequency due to material perturbations, but is not in a very versatile form since in general the $\vec{E}$, $\vec{H}$ is unknown to us. However, if we assume that Δ𝜖 and Δ𝜇 are relatively small, we can approximate the perturbed fields $\vec{E}$,$\vec{H}$ by the original fields $\vec{E_{0}^{*}}$, $\vec{H_{0}}$and 𝜔 in the denominator of (13) by 𝜔_0_, to give the approximate fractional change in the resonant frequency as

$$\begin{aligned} \frac{\omega-\omega_{0}}{\omega_{0}}\approx\frac{-\int_{V_{0}} \left( \Delta\epsilon\left| \vec{E}_{0} \right|^{2}+\Delta\mu\left| \vec{H}_{0} \right|^{2} \right)dv}{\int_{V_{0}} \left( \epsilon\left| \vec{E}_{0} \right|^{2}+\mu\left| \vec{H}_{0} \right|^{2} \right)dv}\#\left( 13 \right) \end{aligned}$$

This result shows that any increase in 𝜖 or 𝜇 at any position in the cavity will decrease the resonant frequency. The reader may also observe that the terms in (13) can be related to the stored electric and magnetic energies in the original and perturbed cavities, so that the decrease in resonant frequency can be related to the increase in stored energy of the perturbed cavity

Due to the Hermitian nature of the eigenvalue equations, the unperturbed cavity modes possess completeness. The perturbed filed modes can be expressed as superposition of the unperturbed filed modes:

$$\begin{aligned} \vec{E}=\sum a_{i}\vec{E}_{i}, \vec{H}=a_{i}\vec{H}_{i}\#\left( 14 \right) \end{aligned}$$

The modes $\vec{E},\vec{H}$ satisfy the condition of filed orthogonality and normalization:

$$\begin{aligned} \int\epsilon_{0}\vec{E}_{m}\vec{E}_{i}^{*}+\mu_{0}\vec{H}_{m}\vec{H}_{i}^{*}=\delta_{mn}\#\left( 15 \right) \end{aligned}$$

Plug this condition into the cavity perturbation formula:

$$\begin{aligned} \frac{\omega-\omega_{0}}{\omega}=\frac{-\int\Delta\epsilon\vec{E}\cdot\vec{E}_{0}^{*}dv}{\int\left( \epsilon_{0}\vec{E}\cdot\vec{E}_{0}^{*}+\mu_{0}\vec{H}\cdot\vec{H}_{0}^{*} \right)dv}=\frac{-\int\Delta\epsilon\vec{E}\cdot\vec{E}_{0}^{*}dv}{a_{0}}\#\left( 16 \right) \end{aligned}$$

Let $\int\Delta\epsilon\vec{E_{i}}\cdot\vec{E_{0}^{*}\text{d}}v=b_{i0}$, so we have

$$\begin{aligned} \int\Delta\epsilon\vec{E}\cdot\vec{E}_{0}^{*}dv=\sum a_{i}\int\Delta\epsilon\vec{E}_{i}\cdot\vec{E}_{0}^{*}dv=\sum a_{i}b_{i0}\#\left( 17 \right) \end{aligned}$$

which simplifies the cavity perturbation formula into:

$$\begin{aligned} \frac{\omega-\omega_{0}}{\omega}=\frac{-\sum a_{i}b_{i0}}{a_{0}}=\frac{-\sum a_{i}b_{i0}}{\sum a_{i}\delta_{i0}}\#\left( 18 \right) \end{aligned}$$

which is:

$$\begin{aligned} \left( \omega-\omega_{0} \right)\sum a_{i}\delta_{i0}=-\omega\sum a_{i}b_{i0}\#\left( 19 \right) \end{aligned}$$

That is:

$$\begin{aligned} \omega\sum a_{i}\left( b_{i0}+\delta_{i0} \right)-\sum\omega_{0}a_{i}b_{i0}=0\#\left( 20 \right) \end{aligned}$$

So, we transform the formula into linear eigen-value problems:

$$\begin{aligned} \left( \begin{matrix} \omega_{i} & 0 & \cdots\\ 0 & \omega_{2} & \cdots\\ \vdots& \vdots& \ddots\end{matrix} \right)\left( \begin{matrix} a_{1} \\ a_{2} \\ \vdots\end{matrix} \right)=\omega\left( \begin{matrix} 1+b_{11} & b_{12} & \cdots\\ b_{21} & 1+b_{22} & \cdots\\ \vdots& \vdots& \ddots\end{matrix} \right)\left( \begin{matrix} a_{1} \\ a_{2} \\ \vdots\end{matrix} \right)\#\left( 21 \right) \end{aligned}$$

The matrix can be further approximated by considering the perturbed modes, which can be approximated as a superposition of the degenerate modes from the unperturbed case at the nearest frequencies. In other words, only the modes corresponding to a single frequency are retained.

$$\begin{aligned} \omega_{0}\left( \begin{matrix} a_{1} \\ a_{2} \\ \vdots\end{matrix} \right)=\omega\left( \begin{matrix} 1+b_{11} & b_{12} & \cdots\\ b_{21} & 1+b_{22} & \cdots\\ \vdots& \vdots& \ddots\end{matrix} \right)\left( \begin{matrix} a_{1} \\ a_{2} \\ \vdots\end{matrix} \right)\#\left( 22 \right) \end{aligned}$$

As for QVHE, we define degenerate eigen-modes $\vec{e_{L}}$, $\vec{e_{R}}$ at K point in the momentum space as the basis, performing a rotation symmetrical analysis, the system is invariant in $\mathcal{R}_{3}$ rotation thus satisfy:

$$\begin{aligned} b_{mn}=\mathcal{R}_{3}b_{mn}\#\left( 23 \right) \end{aligned}$$

where:

$$\begin{aligned} b_{12}=\int\Delta\epsilon\vec{e}_{L}\cdot\vec{e}_{R}^{*}dv=\Delta\epsilon\mathcal{R}_{3}\vec{e}_{L}\cdot\mathcal{R}_{3}\vec{e}_{R}^{*}dv\#\left( 24 \right) \end{aligned}$$

with $\mathcal{R}_{3}\vec{e_{L}}=\text{e}^{\text{i2}\text{/}\text{3π}}\vec{e_{L}},$ $\mathcal{R}_{3}\vec{e_{R}}=\text{e}^{\text{-i2}\text{/}\text{3π}}\vec{e_{R}}$, so we get $b_{12}=e^{i\frac{4}{3}}b_{12}$ that $b_{12}=b_{12}^{*}=0$ which simplifies the matrix to:

$$\begin{aligned} \omega_{0}\left( \begin{matrix} a_{1} \\ a_{2} \end{matrix} \right)=\omega\left( \begin{matrix} 1+b_{11} & 0 \\ 0 & 1+b_{22} \end{matrix} \right)\left( \begin{matrix} a_{1} \\ a_{2} \end{matrix} \right)\#\left( 25 \right) \end{aligned}$$

Near Dirac point 𝐾 and 𝐾′, we encapsulate two Valley in a vector as 𝜓 = (𝑈_𝐾_, 𝑇𝑈_𝐾_′), where $U_{K\left( K^{'} \right)}=\left( \begin{matrix} a_{K\left( K^{'} \right)}^{R},a_{K\left( K^{'} \right)}^{L} \end{matrix} \right)$. Thus the effective Hamiltonian can be written as:

$$\begin{aligned} H_{0}\left( \delta k \right)=\nu_{D}\left( \delta k_{x}\hat{\tau}_{z}\hat{\sigma}_{x}+\delta k_{y}\hat{\tau}_{0}\hat{\sigma}_{y} \right)\#\left( 26 \right) \end{aligned}$$

The perturbation terms introduced by the rotation of the dielectric rods can be written as

$$\begin{aligned} H_{p}=\Delta\omega_{1}^{2}\left( \theta\right)\hat{\tau}_{0}\hat{\sigma}_{z}+\Delta\omega_{2}^{2}\left( \theta\right)\hat{\tau}_{0}\hat{\sigma}_{0}\#\left( 27 \right) \end{aligned}$$

where $\Delta\omega_{1}^{2}\left( \theta\right)=\frac{1}{2}\omega_{0}^{2}\left( \left( 1+b_{11}\left( \theta\right) \right)^{2}-\left( 1+b_{22}\left( \theta\right) \right)^{2} \right)$,$\Delta\omega_{2}^{2}\left( \theta\right)=\frac{1}{2}\omega_{0}^{2}\left( \left( 1+b_{11}\left( \theta\right) \right)^{2}+\left( 1+b_{22}\left( \theta\right) \right)^{2} \right)$, thus the eigen-function can be written as:

$$\begin{aligned} H\left( \delta k \right)\psi=\left( H_{0}\left( \delta k \right)+H_{p} \right)\psi=\left( \omega^{2}\left( \delta k \right)-\omega_{0}^{2} \right)\psi\#\left( 28 \right) \end{aligned}$$

For Pseudo spin hall effect, with the basis (|𝑑_+_⟩, |𝑝_+_⟩, |𝑑_−_⟩, |𝑝_−_⟩), near the Γ point, the effective Hamiltonian can be written as:

$H_{p,d\left( k \right)}=\left( \begin{matrix} \omega_{d}^{2}\left( \theta\right)-\omega_{0}^{2} & \gamma^{*}\left( k_{x}+\text{i}k_{y} \right) & 0 & 0 \\ \gamma\left( k_{x}-\text{i}k_{y} \right) & \omega_{p}^{2}\left( \theta\right)-\omega_{0}^{2} & 0 & 0 \\ 0 & 0 & \omega_{d}^{2}\left( \theta\right)-\omega_{0}^{2} & \gamma^{*}\left( k_{x}-\text{i}k_{y} \right) \\ 0 & 0 & \gamma\left( k_{x}+\text{i}k_{y} \right) & \omega_{p}^{2}\left( \theta\right)-\omega_{0}^{2} \end{matrix} \right)$ (29)

Where 𝛾 is pure imaginary, 𝜔_0_ is the gap-closing frequency. 𝜔_𝑑_ and 𝜔_𝑝_ can be solved using the cavity perturbation theory

$$\begin{aligned} \omega_{0}\left( \begin{matrix} a_{1} \\ a_{2} \\ a_{3} \\ a_{4} \end{matrix} \right)=\omega\left( \begin{matrix} 1+b_{11}\left( \theta\right) & b_{12}\left( \theta\right) & b_{13}\left( \theta\right) & b_{14}\left( \theta\right) \\ b_{21}\left( \theta\right) & 1+b_{22}\left( \theta\right) & b_{23}\left( \theta\right) & b_{24}\left( \theta\right) \\ b_{31}\left( \theta\right) & b_{32}\left( \theta\right) & 1+b_{33}\left( \theta\right) & b_{34}\left( \theta\right) \\ b_{41}\left( \theta\right) & b_{42}\left( \theta\right) & b_{43}\left( \theta\right) & 1+b_{44}\left( \theta\right) \end{matrix} \right)\left( \begin{matrix} a_{1} \\ a_{2} \\ a_{3} \\ a_{4} \end{matrix} \right)\#\left( 30 \right) \end{aligned}$$

Thus giving the effective Dirac Hamiltonian near Γ point:

$$\begin{aligned} H_{p,d\left( k \right)}\Psi=\left( \omega^{2}\left( \delta k \right)-\omega_{0}^{2} \right)\Psi\#\left( 31 \right) \end{aligned}$$

**Section C. Band structures for TPPC under different oriented angles (Figure. S2).**

**Section D. Simulated field distribution for edge states of other configuration combining VPC_1,2_ and PPC (Figure. S3)**

**Section E. Simulation results for HTP-PICS induced by OPC and VPC coupling**

The hybrid topological monocrystalline insulator (HTP-PICS) composed by VPC surrounded by the OPC is depicted in the Figure. S4. The inset in the upper left corner represents the calculated localized quality factor *Q^L^*, illustrated through a colorful map. In contrast, the lower right corner of the inset showcases the distribution of intrinsic states corresponding to corner states. Diverging from the main text, the vicinity of the domain walls does not exhibit notable fractional charges. Consequently, we note that under these circumstances, hybrid edge states are absent. However, the presence of corner states still persists.

**Section F. Comparison of fractional spectral charge in non-hybrid high-order PhC and HTP-PICS**

As shown in Figure. S5, the four types of valley photonic crystals exhibit C_3_ symmetry, enabling the construction of C_3_ symmetric topological insulators. Based on the eigenvalues of the C_3_ operator at high symmetric points in the first Brillouin zone, the bulk polarization and the secondary topological indices are obtained as follows:

$$\begin{aligned} \boldsymbol{P}^{\left( 3 \right)}=\frac{2e}{3}\left( \left[ K_{1}^{\left( 3 \right)} \right]+2\left[ K_{2}^{\left( 3 \right)} \right] \right)\left( \mathbf{a}_{\mathbf{1}}+\mathbf{a}_{\mathbf{2}} \right)\#\left( 32 \right) \end{aligned}$$

$$\begin{aligned} Q_{\text{corner}}^{\left( 3 \right)}=\frac{e}{3}\left( \left[ K_{2}^{\left( 3 \right)} \right] \right) mod e\#\left( 33 \right) \end{aligned}$$

where$\left[ \Pi_{p}^{\left( n \right)} \right]$represents the integer topological invariant, defined as $\left[ \Pi_{p}^{\left( n \right)} \right]\equiv\#\Pi_{p}^{\left( n \right)}-\#\Gamma_{p}^{(n)}$ where$\Pi^{(n)}$refers to high symmetry points of topological insulators with $\Pi^{(n)}$symmetry, and $\#\Pi_{p}^{\left( n \right)}$is the number of energy bands below the bandgap with rotation eigenvalue $\Pi_{p}^{\left( n \right)}=e^{\frac{2\pi i\left( p-1 \right)}{n}}(p=1,2,\cdots n)$. For the considered bandgap, the primitive generators of VPC_1_ and VPC_2_ is $h_{1b}^{(3)}$and $h_{1c}^{(3)}$, respectively. $h_{1b}^{(3)}$has a Wannier center at the Wyckoff positions b in Fig. S5(a), and has topological invariants $\left[ K_{1}^{\left( 3 \right)} \right]=-1$ and $\left[ K_{2}^{\left( 3 \right)} \right]=1$, so we can get the bulk polarization $\boldsymbol{P}=\left( \frac{e}{3},\frac{e}{3} \right)$ and the fractional corner charge $Q_{corner}=\frac{e}{3}$, while $h_{1c}^{(3)}$has its Wannier center at the Wyckoff positions c, and has $\left[ K_{1}^{\left( 3 \right)} \right]=-1$, $\left[ K_{2}^{\left( 3 \right)} \right]=0,$leading to the bulk polarization $\boldsymbol{P}=\left( \frac{e}{3},\frac{e}{3} \right)$ and a zero fractional corner charge, as depicted in Fig. S5(b). The distribution of Wannier centers of OPC and PPC is illustrated in Fig. S5(c), and by counting the portion of bulk Wannier orbitals falling into the corner unit cells, we find that the they do not possess fractional corner charges.

We calculated the " spectral charge" by integrating the local density of states (LDOS) up to the bulk bandgap. Since each band is formed by an individual mode in each unit cell, there are four bands under the considered bandgap, indicating $Q_{bulk}=4$ for the bulk cells. We calculate the spectral charge for the corner cells of VPCs with C_3_ symmetry, getting the value 3.26 for VPC_1_ and 3.02 for VPC_2_, respectively. Due to the lattice's three-fold rotational symmetry, the calculation is performed only for one-third of the structure, and the results are presented in Figure. S6. The fractional parts of the corner spectral charges were approximately 1/3 and 0, which are consistent with the theoretical results get from the distribution of Wannier centers, with errors arising from the finite intra-cell coupling.

The Wannier center distributions of valley photonic crystal (VPC) demonstrated above correspond to the ideal cases in the extremely dimerized limits, where coupling of next-nearest neighbors is neglected, and the ratio between intra- and inter-cell hopping rates $t_{0}$is equal to zero. However, in our model, $t_{0}$ is a finite value. It is worth noting that the decay of the fractional corner charge $Q\left( r \right)$ should follow an exponential law $Q\left( r \right)\propto e^{-\alpha\left| r \right|}$, where $\alpha$depends on $t_{0}$. In the extremely dimerized limits, where$t_{0}=0$, the fractional charge is localized entirely at the corner unit cell of the lattice, and $\alpha\to\infty$. As the intra-cell hopping rate increases, the electrons penetrate into the bulk from the corner unit cell due to the tunneling effect, and $\alpha$ becomes a finite value. The calculation results of the bulk and edge charges presented in Figure. S6(a) also show a certain degree of charge diffusion, with the bulk unit cells far from the boundaries and corners having spectral charge closest to the integer 4.

When the VPC is surrounded by pseudospin photonic crystal (PPC), their different valley Chern numbers support the existence of topological edge states. However, due to the distinct symmetries (C_3_ and C_6_) of these two lattices, the boundary symmetry is reduced. Consequently, the boundary no longer follows the extremely dimerized limits, and the next-nearest-neighbor interactions become significant, leading to the shift of the Wannier centers. As a result, it becomes challenging to predict the existence of corner states by calculating the fractional charge of corner cells based solely on the distribution of the Wannier centers. Nevertheless, we can still predict the existence of corner states by calculating the fractional value of spectral charge. Through calculations, we find that the spectral charge of the corner unit cells changes due to the break of symmetry, with a fractional value being approximately 2/3, as shown in Figure. S7. Upon examining the simulation results, we observe a certain degree of hybridization between bulk states, edge states, and corner states near the bandgap. While careful discrimination is made, a small contribution from localized states cannot be completely avoided when calculating the corner spectral charge. Nevertheless, it is worth noting that the significant fractional value still signifies the presence of corner states within the bandgap.

We find that the existence of corner states is related to the topological domain walls through research. When two C_3_ symmetric VPC lattices are enclosed by PPC lattice, different topological domain walls are formed, as shown in Figure. S7.

**Section G. Snapshots of fabricated samples**

The snapshot of samples is shown in Figure. S8.

**Section H. The measured positions of transmission intensity**

As depicted in Figure. S9, the local field intensity at points in Figure 3h and Figure 5e in the main text are determined by the mean value obtained from experimental data at five adjacent spatial points surrounding the dielectric ellipse.

**Section I. Simulation results of topological states for HTP-PICS (Figure. S10)**

**Section J. Absence of 120 ° corner states in HTP-PICS**

In Figure. S11(a), two C_3_ symmetric lattices composed of unit cells of VPC_1_ with △*θ* = 45° and VPC_2_ with △*θ* =135°, respectively, are surrounded by PPC lattice, while in Fig. S11(b), the triangular lattice is composed of unit cells of VPC_2_ with △*θ* = 45° and 135°. In both models, corner states only exist at the 60° splicing corners, while being absent at 120° splicing angles. According to the interaction between valleys, the sign flip of valley Chern numbers is identified as a necessary condition for the formulation of corner states. In Fig. S11a, each 60° splicing angle is formed by two identical topological domain walls, connecting edge states belonging to different valleys with opposite valley Chern numbers. While at the 120° splicing angle, there is a disorder in the connection of topological domain walls, breaking their continuity, which cannot support corner states. In Fig. S11b, the configuration of domain walls at the 60° splicing angle is the similar to that in Fig. S11a, However, for the 120° splicing angles, two different topological domain walls with the same valley Chern number are connected, which is impossible for corner states to exist.

**Section K. Measurement of relative dielectric constant and magnetic permeability**

The photonic crystalline rods consist of a composite material of polyphenylene ether (PPE) and ceramic. The dielectric constant and magnetic permeability of this material were measured using the transmission line method. As illustrated in the Figure. S12a, the specimen was placed into a rectangular waveguide (KEYSIGHT X11644A). The *S* parameters of the transmission line were measured using a network analyzer (KEYSIGHT N5224B). The dielectric constant and magnetic permeability of the sample were determined through the *S*-parameter retrieval method. Following the measurements, Figure. S12b prove that the relative dielectric constant of the material remained stable within the range of 9.350 to 12.194 over the frequency range of 8.200 GHz to 9.000 GHz. Similarly, within the same frequency range, the relative magnetic permeability of the material exhibited stability within the range of 0.9547 to 1.0715.


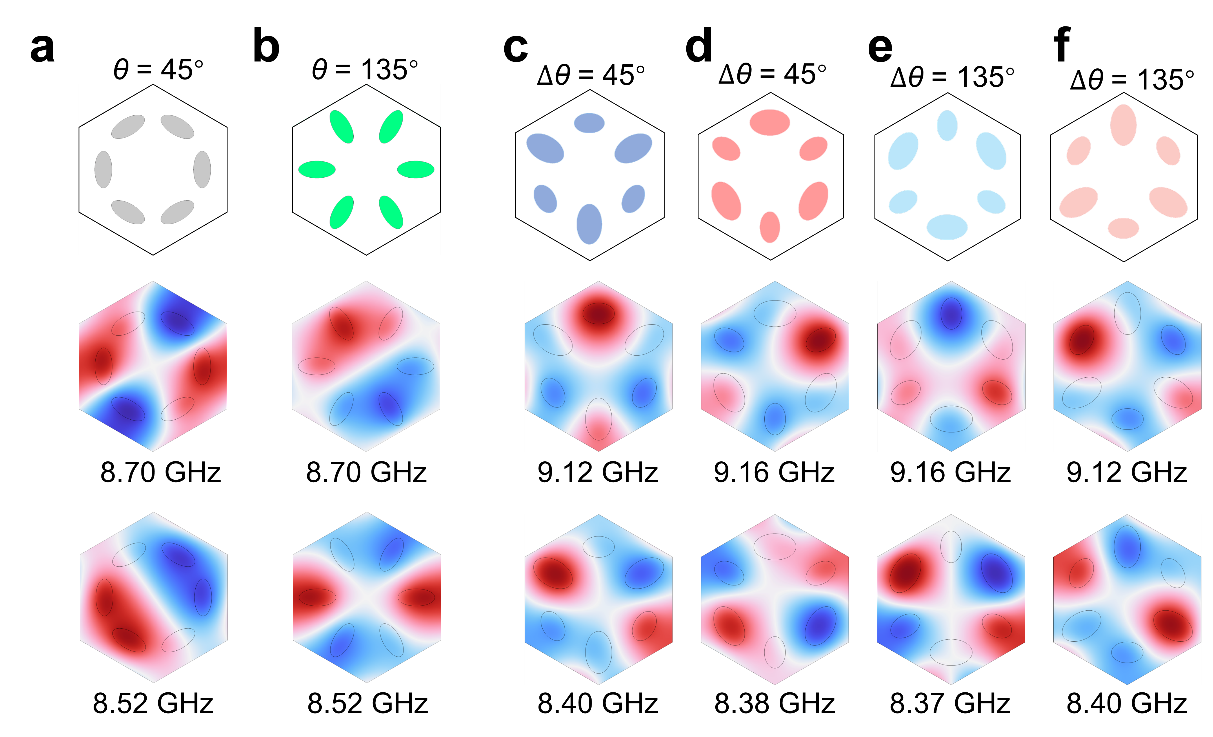


**Fig. S1. Calculated eigen-modes of photonic lattices. a**,**b** configuration and eigenmodes for OPC and PPC lattice. **c-f.** different VPC configurations along with their corresponding eigenmodes at high and low frequencies.


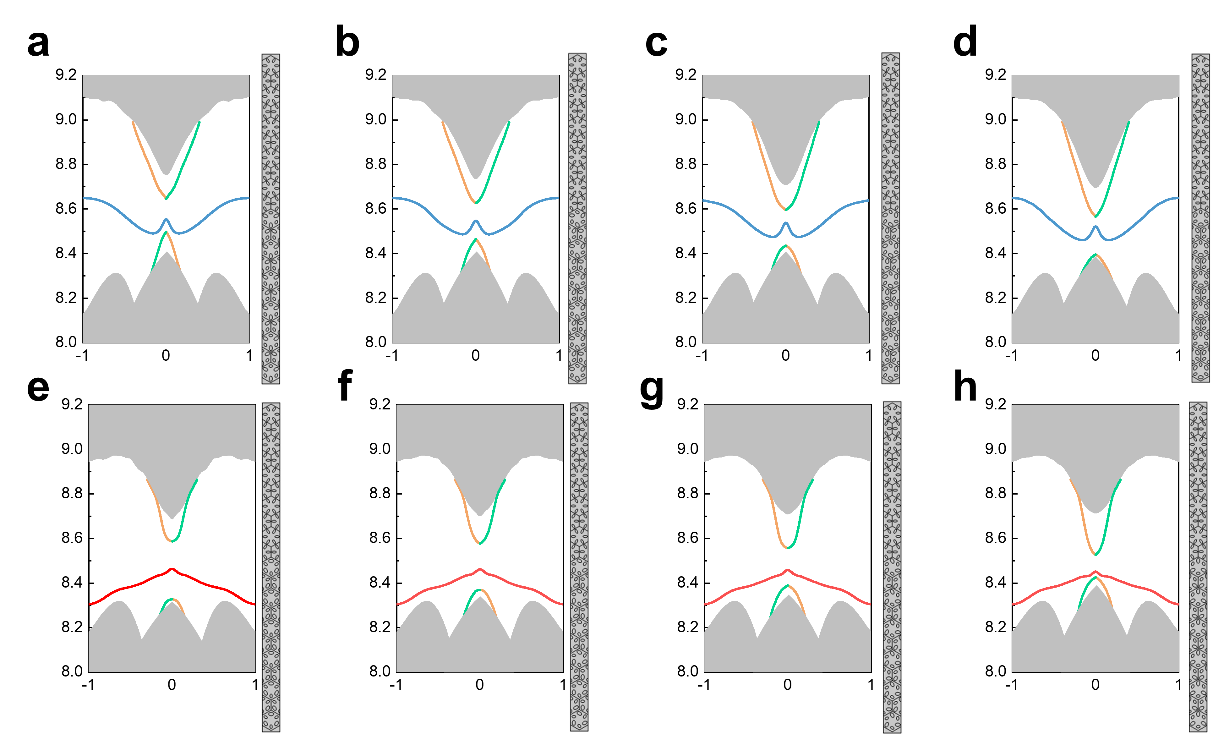


**Fig. S2. Band structures for TPPCs under different oriented angles.** (a)-(d) VPC_1_-PPC configuration. (e)-(h) VPC_2_-PPC configuration.


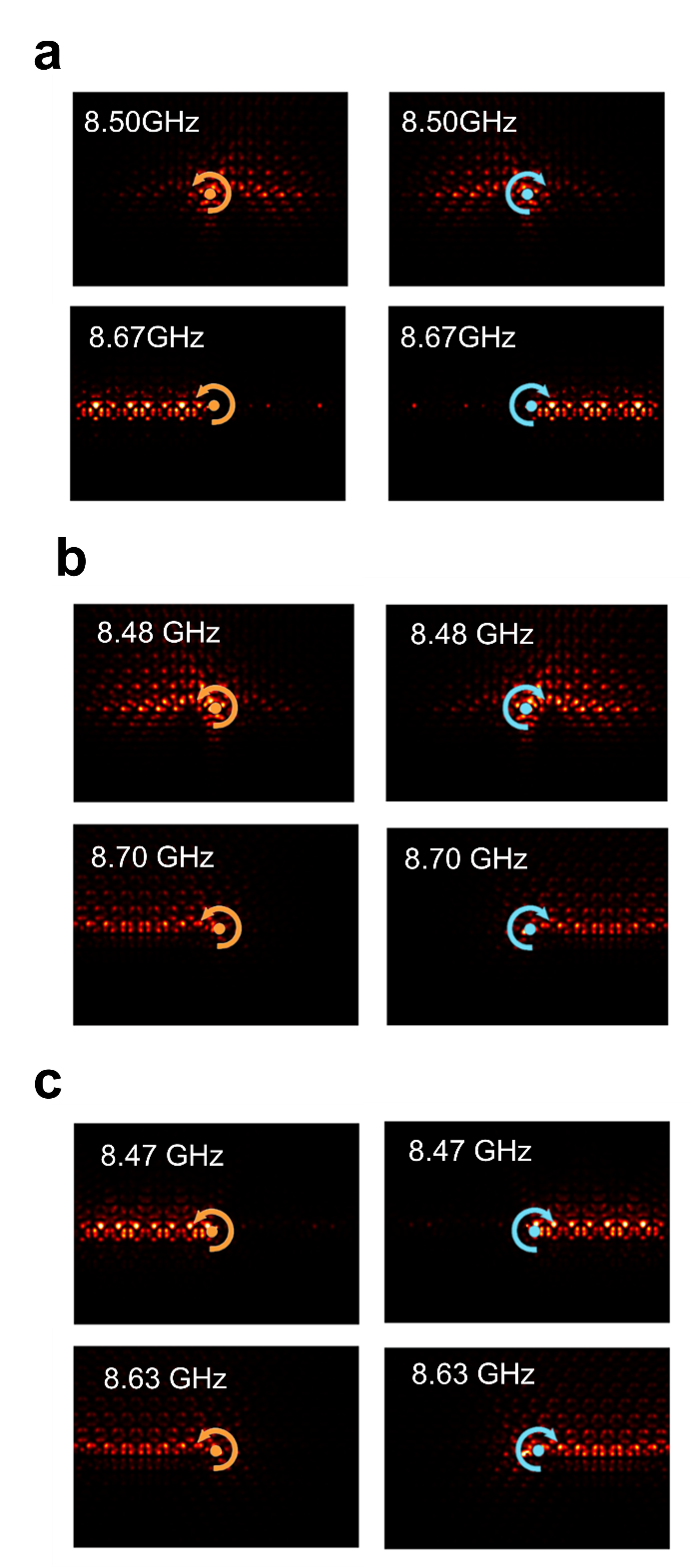


**Fig. S3. Simulated field distribution for edge states of another configuration combining VPC1,2 and PPC.** From **a** to **c**, the interfaces are composed by PPC and VPC_1_ with △*θ* = 135°, VPC_2_ with △*θ* = 45° and VPC_2_ with △*θ* =135°, respectively.


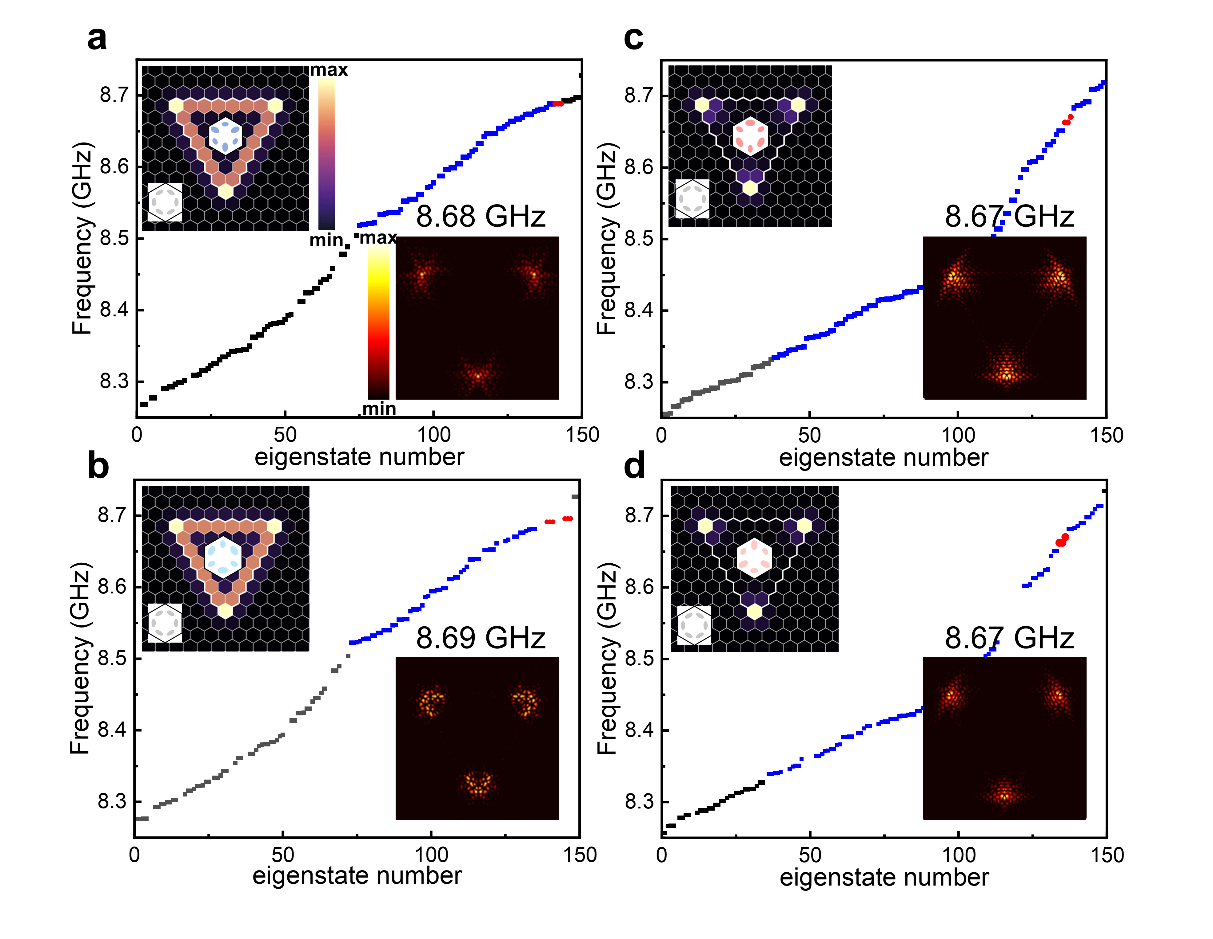


**Fig. S4.**

**Eigenstates for OPC-VPC coupled HTP-PICS.** From **a** to **d**, the enclosed VPC lattices are VPC_1_ with △*θ* = 45° , 135° and VPC_2_ with △*θ* = 45° , 135°, the upper left panel is the *Q^L^* distribution, and the lower right panel is the field distribution of the second-order eigenstate.


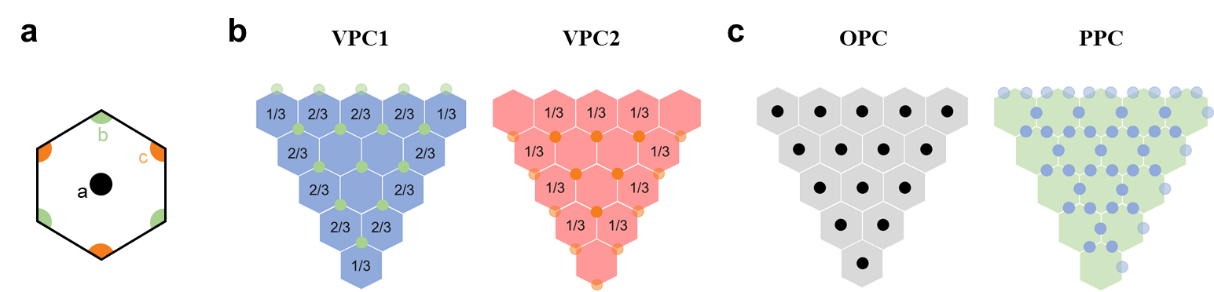


**Fig. S5.**

**Edge and corner fractional charges for photonic crystals (PhCs). a**. Maximal Wyckoff positions for C_3_-symmetric unit cells. **b**. Edge and corner fractional charges for VPC_1_ and VPC_2_ with Wannier centers at maximal Wyckoff positions. **c**. The distribution of Wannier centers at maximal Wyckoff positions for C_6_-symmetric unit cells, showing no fractional edge and corner charges both for OPC and PPC.


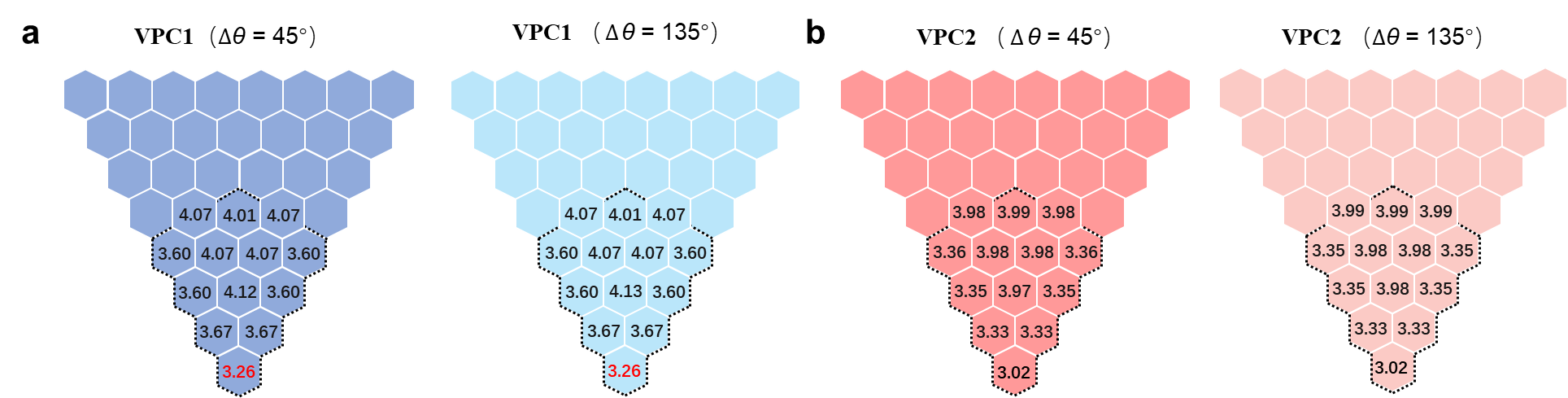


**Fig. S6.**

**Corner spectral charge for VPCs. a.** the distribution of spectral charge for VPC_1_ with △*θ* = 45°, 135°, and the corner unit cells have a fractional value of 1/3 approximately. **b.** The distribution of spectral charge for VPC_2_ with △*θ* = 45°, 135° with no fractional corner charge.


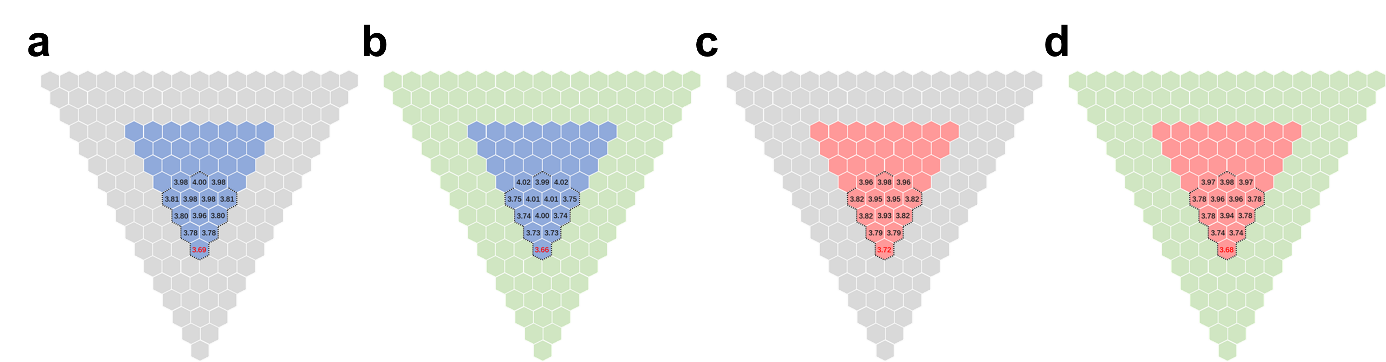


**Fig. S7.**

**Corner spectral charge at the splicing interfaces of hybrid PCs. a-b** The spectral charge distribution of VPC_1_ surrounded by OPC (**a**) and PPC (**b**). **c-d** The spectral charge distribution of VPC_2_ surrounded by OPC (**c**) and PPC (**d**).


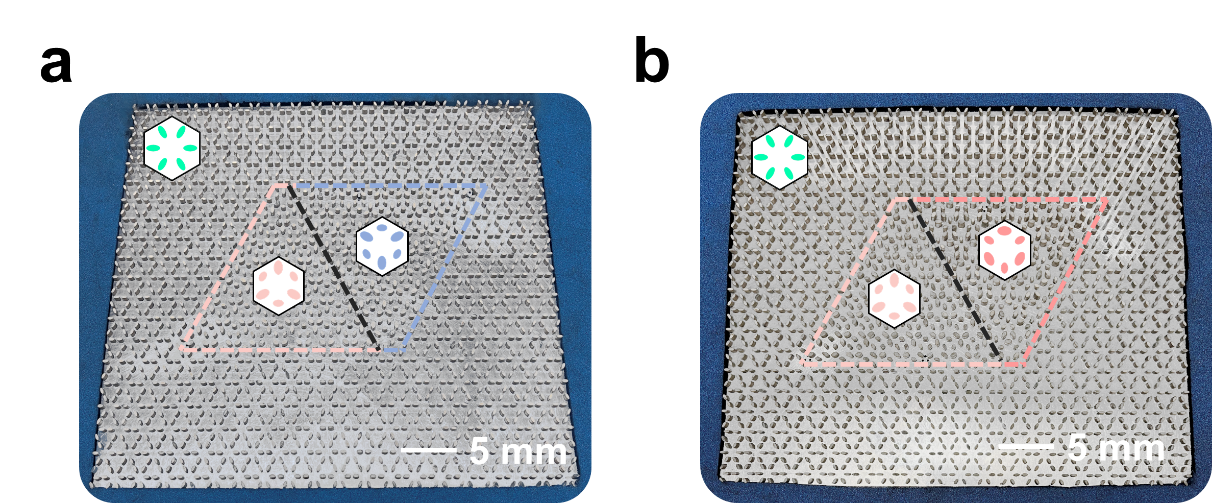


**Fig. S8.**

**The snapshots of fabricated HTP-PICS.** Each sample is glued to a metal plate by dielectric pillars with a scale bar of 5 mm. The HTP-PICSs are formed by **a**. PPC-VPC1(△*θ* = 45°)-VPC2(△*θ* = 135°), **b**. PPC-VPC2(△*θ* = 45°)-VPC2(△*θ* = 135°).


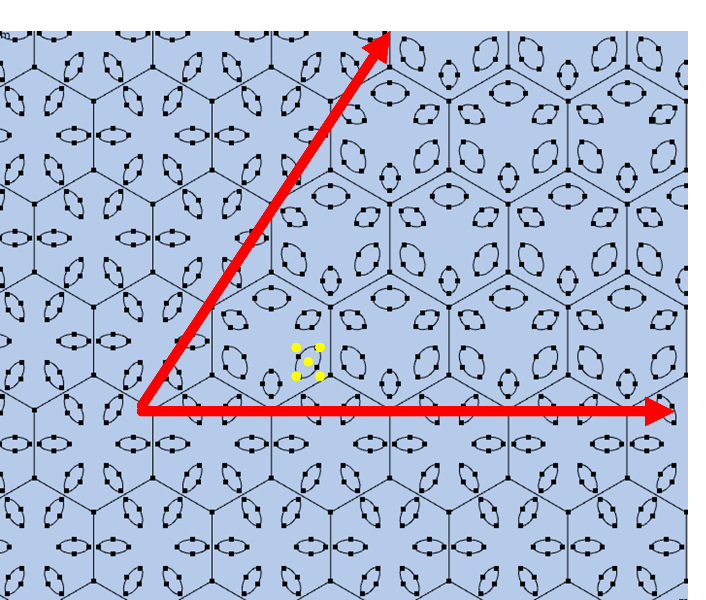


**Fig. S9.**

**The measured positions of the intensity spectrum.** The mean value of the data collected from five distinct locations around a single dielectric ellipse has been taken to obtain the final transmission intensity.


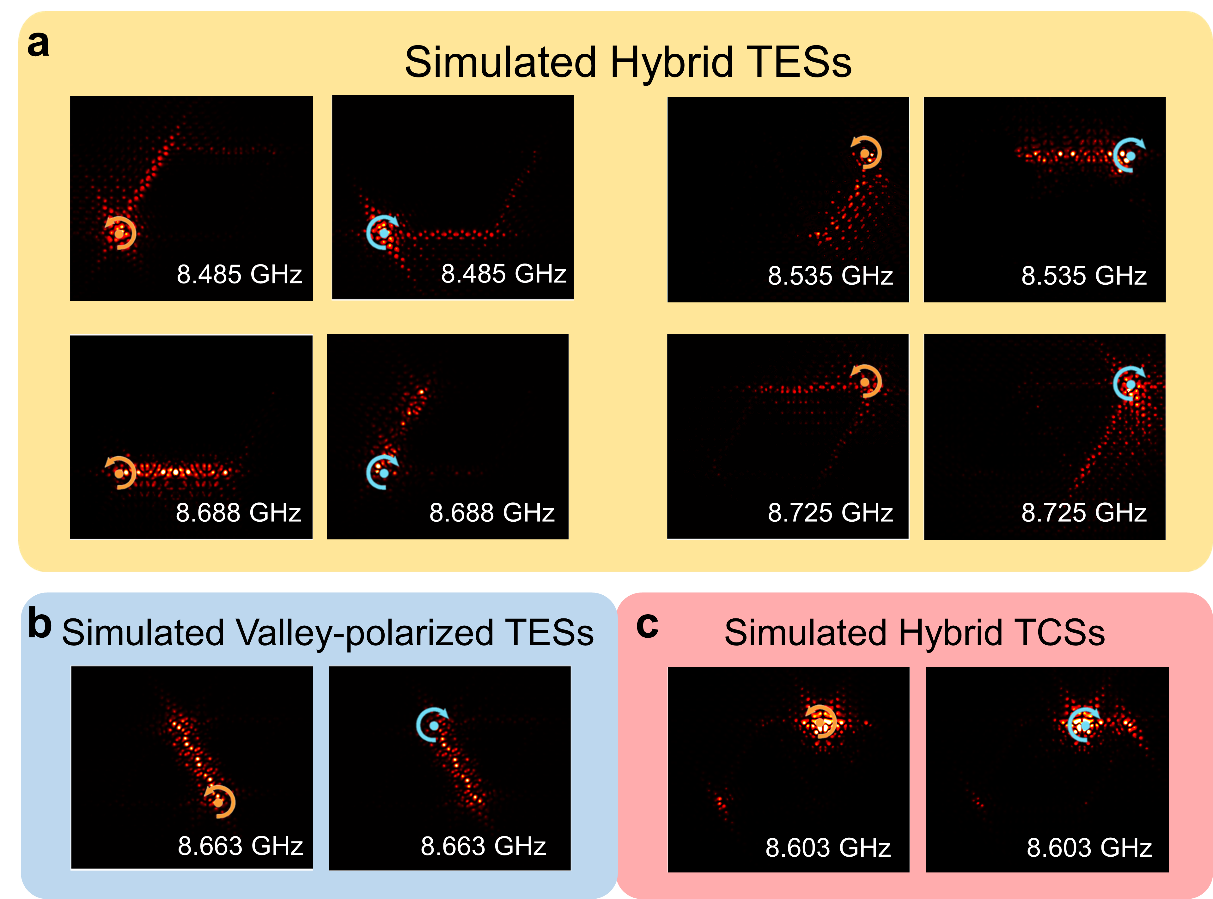


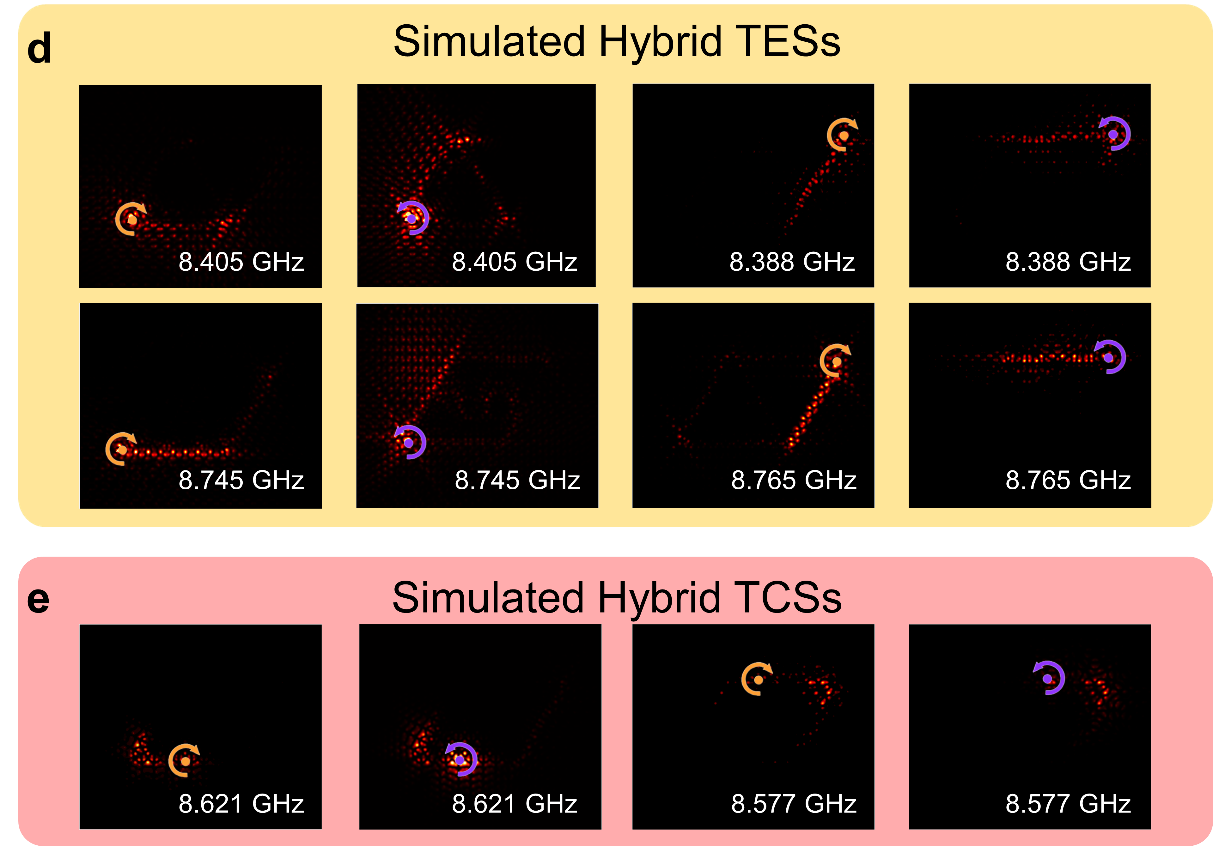


**Fig. S10.**

**Simulation results of high-order HTP-PICSs. a-c.** Simulated hybrid topological modes corresponding to Fig. 3 in the main text, where **b** is the valley-associated first-order mode, and **c** is the frequency-maintained corner mode. **d-e.** Simulated hybrid topological states corresponding to Fig. 4, where **d** represents the hybrid edge modes, and **e** is the frequency-dependent corner modes.


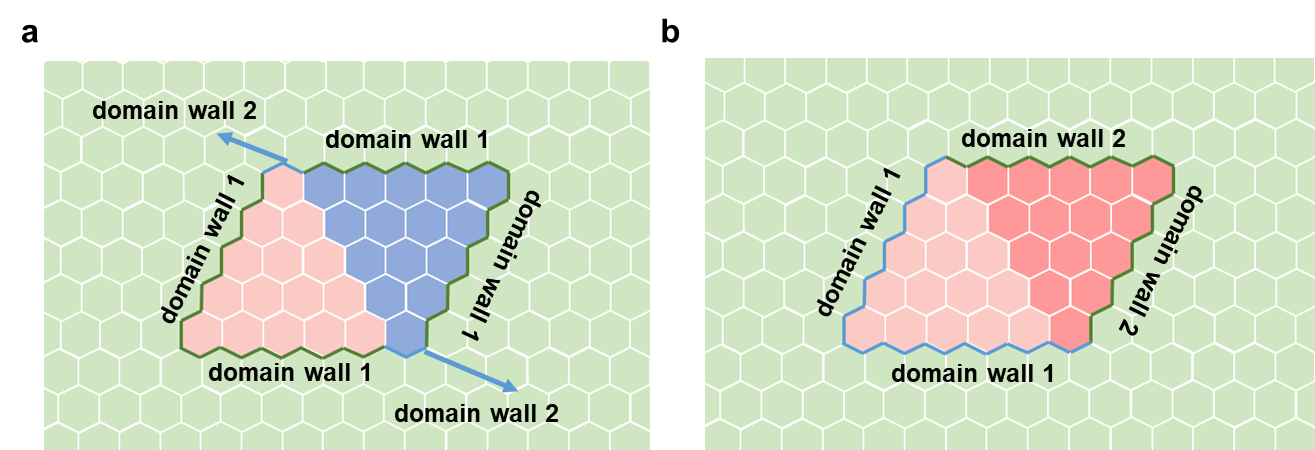


**Fig. S11.**

**Topological domain walls in HTP-PICSs. a.** The hybrid PhC formed by VPC_1_ with △*θ* = 45° and VPC_2_ with △*θ* =135° surrounded by PPC with multiple segments of topological domain walls. **b.** The hybrid PhC formed by VPC_2_ with △*θ* = 45° and 135° surrounded by PPC, exhibiting two kinds of continuous topological domain walls.


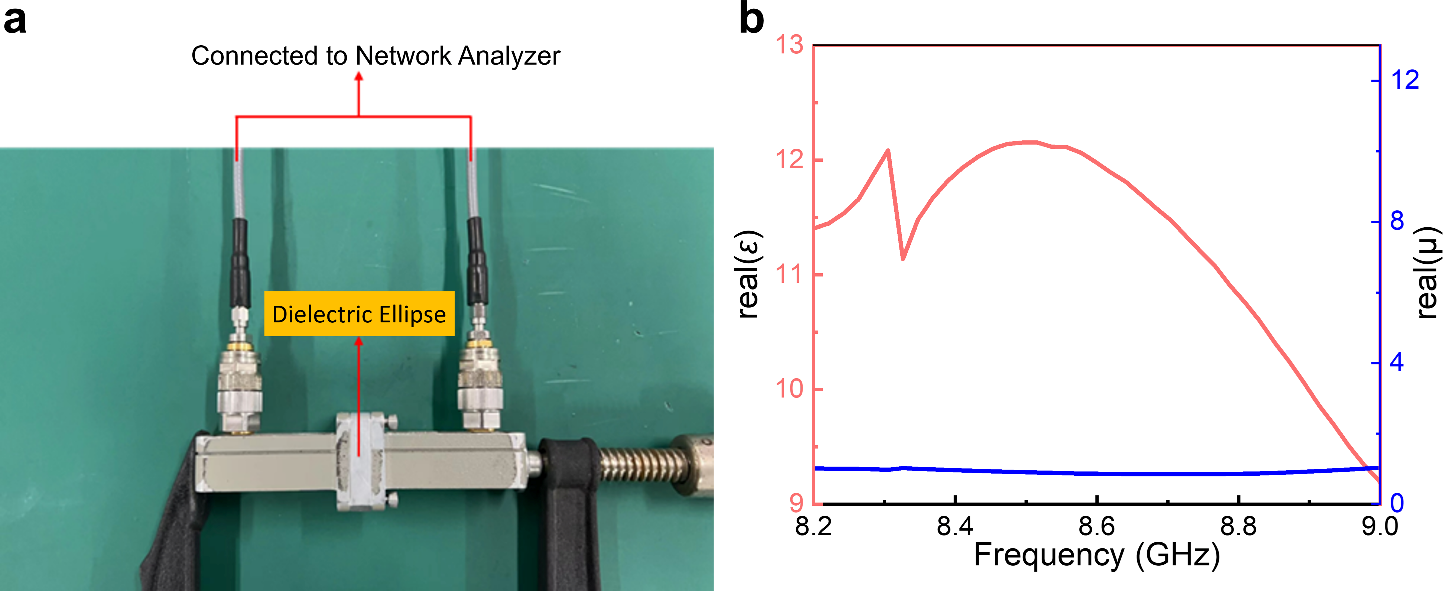


**Fig. S12.**

**Material property measurement. a**. experimental setup. **b**. measurement result of relative dielectric constant and magnetic permeability as a function of frequency.
